# Roles of long noncoding RNAs in human inflammatory diseases

**DOI:** 10.1038/s41420-024-02002-6

**Published:** 2024-05-15

**Authors:** Yuliang Zhang, Hongliang Liu, Min Niu, Ying Wang, Rong Xu, Yujia Guo, Chunming Zhang

**Affiliations:** 1 Shanxi Key Laboratory of Otorhinolaryngology Head and Neck Cancer, First Hospital of Shanxi Medical University, Taiyuan, 030001 Shanxi China; 2 Shanxi Province Clinical Medical Research Center for Precision Medicine of Head and Neck Cancer, First Hospital of Shanxi Medical University, Taiyuan, 030001 Shanxi China; 3 Department of Otolaryngology Head & Neck Surgery, First Hospital of Shanxi Medical University, Taiyuan, Shanxi 030001 China

**Keywords:** Mechanisms of disease, Long non-coding RNAs

## Abstract

Chemokines, cytokines, and inflammatory cells mediate the onset and progression of many diseases through the induction of an inflammatory response. LncRNAs have emerged as important regulators of gene expression and signaling pathways. Increasing evidence suggests that lncRNAs are key players in the inflammatory response, making it a potential therapeutic target for various diseases. From the perspective of lncRNAs and inflammatory factors, we summarized the expression level and regulatory mechanisms of lncRNAs in human inflammatory diseases, such as cardiovascular disease, osteoarthritis, sepsis, chronic obstructive pulmonary disease, asthma, acute lung injury, diabetic retinopathy, and Parkinson’s disease. We also summarized the functions of lncRNAs in the macrophages polarization and discussed the potential applications of lncRNAs in human inflammatory diseases. Although our understanding of lncRNAs is still in its infancy, these data will provide a theoretical basis for the clinical application of lncRNAs.

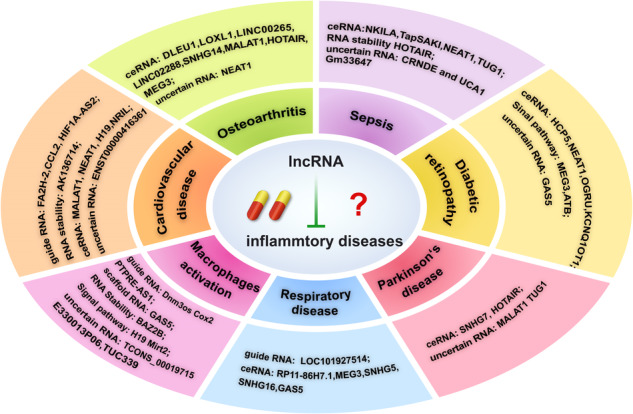

## Facts

- Inflammatory response caused by chemokines, cytokines, and inflammatory cells mediates the onset and progression of many diseases.
- LncRNAs are a class of RNA molecules that are longer than 200 nucleotides that control inflammation-related gene expression at chromatin modification, mRNA stability, miRNA sponge, and signaling pathways.
- LncRNAs can regulate macrophage polarization.

## Open questions

- What are the mechanisms of lncRNAs and inflammatory factors on human inflammatory diseases?
- How are lncRNAs involved in the progression of human inflammatory diseases?
- How can we target lncRNAs to alleviate inflammatory response in human disease?

## Introduction

Inflammation is an adaptive response triggered by noxious stimuli and conditions, such as infection and tissue injury. Participation of chemokines, cytokines, and different inflammatory cells is required to progress this complex protective mechanism to control harmful factors and eliminate damaged tissues [1]. However, sustained and uncontrolled immune reactions promote chronic inflammation and lead to chronic diseases [2]. Many studies have shown a close link between inflammation and many diseases, including cardiovascular disease, osteoarthritis, sepsis, chronic obstructive pulmonary disease, asthma, acute lung injury, diabetic retinopathy, and Parkinson’s disease. Many studies have shown that chemokines and cytokines involve in the progress of the diseases, such as IL-1β, IL-6, IL-8, IL10, TNF‑α, and other molecules. Despite recognizing the importance of inflammatory dysregulation in chronic diseases, the underlying mechanisms of inflammatory regulation remain poorly understood [3].

**Fig. 1:**
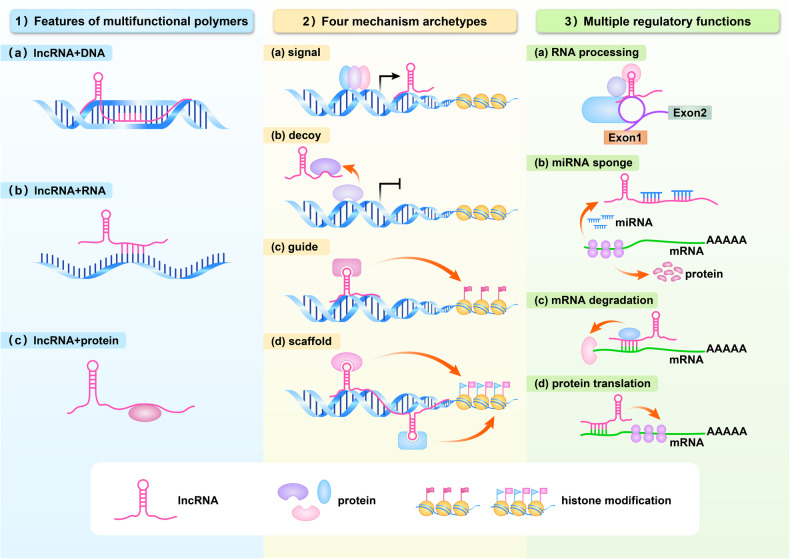
The cellular mechanisms of lncRNAs. 1) LncRNA has a conserved secondary structure and can interact with DNA, RNA, and proteins; 2) LncRNAs are classified as **a** Signal lncRNAs, which are specifically associated with signaling pathways and regulate downstream gene transcription; **b** decoy lncRNAs, which interact with transcription factors and remove them from chromatin; thereby influencing transcriptional regulation; **c** guide lncRNAs, which bind to protein complexes with regulatory effects or enzymatic activities and direct them to target gene promoters or specific genomic sites to regulate downstream signaling events and gene expression; **d** scaffold lncRNAs, a ‘central platform’ connecting various protein complexes, which are directed to a specific genomic location or target gene promoter region to regulate gene expression. 3) LncRNA can participate in the regulation of a variety of biological processes, RNA processing, miRNA sponge (ceRNA), mRNA stability, and protein translation.

Long noncoding RNAs (LncRNAs) have emerged as potential key regulators of the inflammatory response by modulating the transcriptional control of inflammatory genes [4]. LncRNAs are a class of RNA molecules longer than 200 nt believed to be a byproduct of RNA polymerase II transcription with no biological function. However, recent studies have shown that lncRNAs have a conserved secondary structure and can interact with DNA, RNA, and proteins [5, 6]. LncRNAs are classified according to their functions: (a) signal lncRNAs, which are specifically associated with signaling pathways and regulate downstream gene transcription; (b) decoy lncRNAs, which interact with transcription factors and remove them from chromatin; thereby influencing transcriptional regulation; (c) guide lncRNAs, which bind to protein complexes with regulatory effects or enzymatic activities and direct them to target gene promoters or specific genomic sites to regulate downstream signaling events and gene expression; (d) scaffold lncRNAs, a ‘central platform’ connecting various protein complexes, which are directed to a specific genomic location or target gene promoter region to regulate gene expression [7]. Specifically, lncRNAs can regulate a variety of biological processes, including genetic imprinting [8, 9], chromatin modification, RNA processing [10, 11], miRNA sponge [12], mRNA degradation [13], and protein translation [14]. MiRNA sponge, also known as competing endogenous RNA (ceRNA), can regulate the expression of target genes by competitively binding to miRNAs [15] (Fig. 1).

In this review, we summarized the data on the expression level and regulation mechanisms of lncRNAs and inflammatory factors in human inflammatory diseases, focusing on transcription regulation, mRNA stability, miRNA sponge, and signaling pathways. Although our understanding of lncRNAs is still in its infancy, these examples may provide meaningful insights regarding the role of lncRNAs in human inflammatory diseases.

## Roles of lncRNAs in cardiovascular disease

**Fig. 2:**
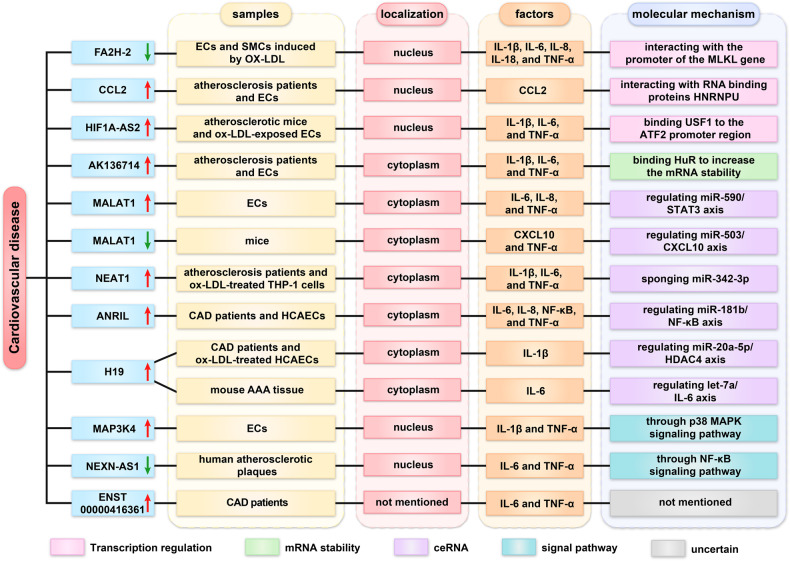
Roles of lncRNA in cardiovascular disease.

Atherosclerosis and coronary artery disease (CAD) are primary inflammatory cardiovascular diseases that have a significant impact on the global health of humans [16, 17]. Recent studies have elucidated the regulatory mechanisms of lncRNAs and inflammatory factors in atherosclerosis and CAD (Fig. 2).

### Transcription regulation

Oxidized LDL (ox-LDL) plays a crucial role in atherosclerosis by acting on multiple cells, such as endothelial cells (ECs), macrophages, and smooth muscle cells (SMCs) [18]. Ox-LDL could stimulate the inflammatory response in ECs and SMCs by increasing the production of VCAM-1 (vascular cell adhesion molecule-1), MCP-1 (monocyte chemotactic protein 1), IL-1β, IL-6, IL-8, IL-18, and TNF-α, while decreasing the levels of lncRNA-FA2H-2. FA2H-2 could downregulate MLKL expression by interacting with the promoter of the MLKL gene. FA2H-2 downregulation or MLKL overexpression can significantly aggravate inflammatory responses. The results suggested that FA2H-2 and MLKL may be potential therapeutic targets in atherosclerosis [19]. Khyzha et al. also found that lncRNA-CCL2 was upregulated in atherogenesis patients and inflammatory ECs. LncRNA-CCL2 increases CCL2 mRNA levels by interacting with the RNA-binding protein HNRNPU, associates with transcription initiation, and promotes vascular inflammation [20]. Additionally, lncRNA HIF1A-AS2 was highly expressed in atherosclerotic mice. HIF1A-AS2 knockdown could attenuate inflammatory response by blocking USF1 binding to the ATF2 promoter region in ox-LDL-exposed ECs, SMCs, and HCAECs [21].

### Regulation of mRNA stability

Numerous RNA-binding proteins (RBPs) may influence the metabolic processes of target RNAs, including splicing, localization, stability, and translation [22]. Human antigen R (HuR) is one of the most studied RBPs with a regulatory impact on RNA metabolism [23]. The lncRNA AK136714 was elevated in the plaque and plasma of the atherosclerosis patients. AK136714 knockdown could decrease IL-1β, IL-6, and TNF-α levels by binding directly to HuR to maintain mRNA stability, thereby protecting the endothelial barrier [24].

### Regulation of miRNA sponge

Overexpression of the lncRNA MALAT1 could promote the production of IL-6, IL-8, and TNF-α through regulation of the miR-590/STAT3 axis, thereby enhancing the inflammatory activities of ECs [25]. However, MALAT1 knockdown could aggravate atherosclerotic lesion formation in mice via regulating miR-503/CXCL10 [26]. This suggested that the same lncRNA has different mechanisms of action in various species. Wang et al. also found that lncRNA NEAT1 was significantly increased in atherogenesis patients and ox-LDL-treated THP-1 cells. NEAT1 knockdown could decrease IL-1β, IL-6, COX2, and TNF-α protein levels by targeting miR-342-3p [27]. Additionally, lncANRIL and H19 were highly expressed in CAD patients. ANRIL promotes the expression of IL‐6, IL‐8, NF‐κB, TNF‐α, ICAM‐1, VCAM‐1, and COX‐2 by regulating miR‐181b/NF‐κB in HCAECs [28]. H19 knockdown alleviated cell inflammation by regulating the miR-20a-5p/HDAC4 axis [29]. Meanwhile, abdominal aortic aneurysm (AAA) is recognized as a chronic vascular inflammatory disease. H19 was upregulated in AAA tissue samples from mice. H19 may promote AAA formation by regulating the let-7a/IL-6 axis [30].

### Regulation of signal pathway

LncRNA-MAP3K4 expression was upregulated in the vessel walls. LncRNA-MAP3K4 knockdown reduced the expression of IL-1β, TNF-α, and COX2 expression through the p38 MAPK signaling pathway in ECs [31]. However, lncRNA NEXN-AS1 was decreased in human atherosclerotic plaques. Overexpression of NEXN-AS1 may inhibit TLR4 oligomerization, the NF-κB pathway, and inflammatory response in ECs [32].

### Uncertain regulatory mechanisms

Li et al. found that lncRNA ENST00000416361 is highly expressed in CAD patients. ENST00000416361 knockdown markedly downregulated IL-6 and TNF‑α levels in human umbilical vein endothelial cells, but the specific underlying mechanism has not been elucidated [33]. In summary, lncRNA could regulate inflammatory factors in atherosclerosis and CAD through complex regulatory mechanisms and serve as a new therapeutic target.

## Roles of lncRNAs in osteoarthritis disease

**Fig. 3:**
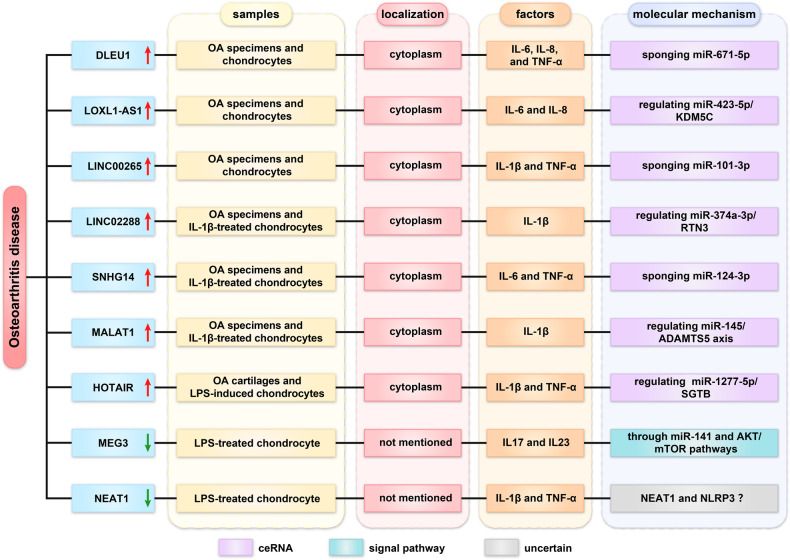
Roles of lncRNA in osteoarthritis disease.

In the elderly, osteoarthritis (OA) is a prevalent degenerative joint disease. The mechanisms of inflammation in bone and joint tissue are complex [34]. Recent research indicates that lncRNA contributes to the development of osteoarthritis (Fig. 3).

### Regulation of miRNA sponge

LncDLEU1, LOXL1-AS1, and LINC00265 were upregulated in OA specimens and OA chondrocytes. DLEU1 could promote the proliferation of chondrocytes and increase the secretion of IL-6, IL-8, and TNF-α by regulating miR-671-5p [35]. LOXL1-AS1 silencing attenuated proliferation and inflammation via targeting miR-423-5p/KDM5C in chondrocytes [36]. Similarly, LINC00265 knockdown inhibited OA chondrocyte apoptosis and inflammation by acting as a miR-101-3p sponge [37].

IL-1β-treated OA chondrocytes were frequently used as a model for chondrocyte injury. LINC02288, lncRNA SNHG14, and MALAT1 were upregulated in OA specimens and IL-1β-treated OA chondrocytes. Linc02288 knockdown significantly reduced the apoptosis of OA chondrocytes and the production of pro-inflammatory cytokines by targeting the miR-374a-3p/RTN3 axis. Similarly, SNHG14 knockdown could inhibit cell apoptosis and decrease COX2, iNOS, TNF-α, and IL-6 expression by targeting miR124-3p [38, 39]. MALAT1 overexpression could modulate IL-1β-induced chondrocyte viability and cartilage ECM degradation by regulating miR-145/ADAMTS5 axis [40]. Additionally, LPS-stimulated chondrocytes were also used as a model for OA. LncRNA HOTAIR was upregulated in OA cartilages and LPS-stimulated CHON-001 chondrocytes. HOTAIR depletion inhibited LPS-induced apoptosis and inflammation by regulating the miR-1277-5p/SGTB pathway [41]. Nevertheless, many low-expressed lncRNAs also play key roles in the development of OA. MEG3 and NEAT1 were downregulated in LPS-treated chondrocytes. MEG3 overexpression resulted in cell proliferation and inhibited inflammation via targeting miR-141 and the AKT/mTOR signaling pathway [42]. NEAT1 can inhibit the expression of inflammatory cytokines, osteogenesis‑related proteins, and NLRP3 [43]. However, the underlying specific mechanism of NEAT1 and NLRP3 in OA has not been elucidated and requires further investigation. In conclusion, lncRNAs can regulate the expression of inflammatory factors and may represent a new therapeutic target in OA.

## Roles of lncRNAs in sepsis

**Fig. 4:**
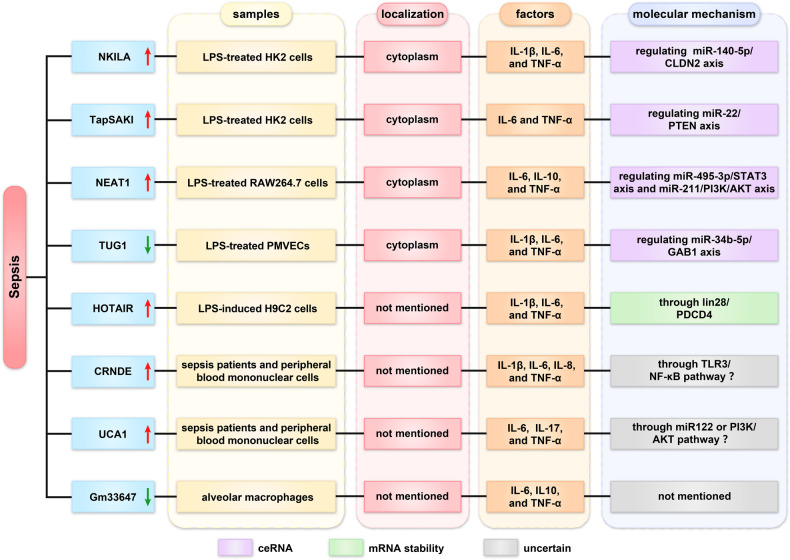
Roles of lncRNA in sepsis disease.

Sepsis is an unusual systemic reaction to a common infection, representing a pattern of immune system response to injury [44]. Increasing evidence suggests that lncRNAs are involved in the development of sepsis (Fig. 4).

### Regulation of miRNA sponge

LPS-treated HK2 cells can generally simulate sepsis-induced AKI. The lncRNA NKILA and TapSAKI were upregulated in LPS-treated HK2 cells. NKILA silencing protected HK2 cells against LPS-induced impairments by regulating the miR-140-5p/CLDN2 axis. TapSAKI knockdown could reduce IL-6 and TNF-α by regulating the miR-22/PTEN axis [45, 46]. Additionally, NEAT1 was upregulated in LPS-treated RAW264.7 cells. Overexpression of NEAT1 may aggravate inflammation by modifying the miR-495-3p/STAT3 and miR-211/PI3K/AKT axes [47]. However, TUG1 was downregulated in LPS-treated PMVECs. Overexpression of TUG1 improved sepsis-induced pulmonary injury, apoptosis, and inflammation via targeting miR-34b-5p/GAB1 [48].

### Regulation of mRNA stability

LIN28 is an RNA-binding protein that participates in many biological processes [49]. Ni et al. found that HOTAIR increased IL-1β, IL-6, and TNF-α levels by binding lin28 to enhance PDCD4 stability in LPS-induced H9C2 cells. HOTAIR knockdown alleviated cardiac function injury and reduced secretion of inflammatory factors in septic cardiomyopathy [50].

### Uncertain regulatory mechanism

Yang et al. found that LncRNA CRNDE and UCA1 were highly expressed in sepsis patients. CRNDE is positively correlated with IL-1β, IL-8, and TNF-α. CRNDE may induce an inflammatory response in sepsis by directly regulating the TLR3/NF‐κB pathway; however, functional experiments are necessary to confirm this hypothesis [51]. UCA1 positively correlates with IL-6, IL-17, and TNF-α, but the exact mechanism is unknown. Wang et al. found that UCA1 directly regulated several miRNAs and pathways, such as miR‐122 and the PI3K/AKT pathway [52, 53]. This implied that the specific mechanism of UCA1 in sepsis still needs validation. Similarly, lncRNA Gm33647 was downregulated in alveolar macrophages. The knockdown of Gm33647 could increase the expression of IL-6, IL10, and TNF-α. The precise functions of Gm33647, however, require further investigation [54]. In summary, lncRNAs can regulate the expression of inflammatory factors and may represent a new therapeutic target in sepsis.

## Roles of lncRNAs in respiratory inflammatory diseases

**Fig. 5:**
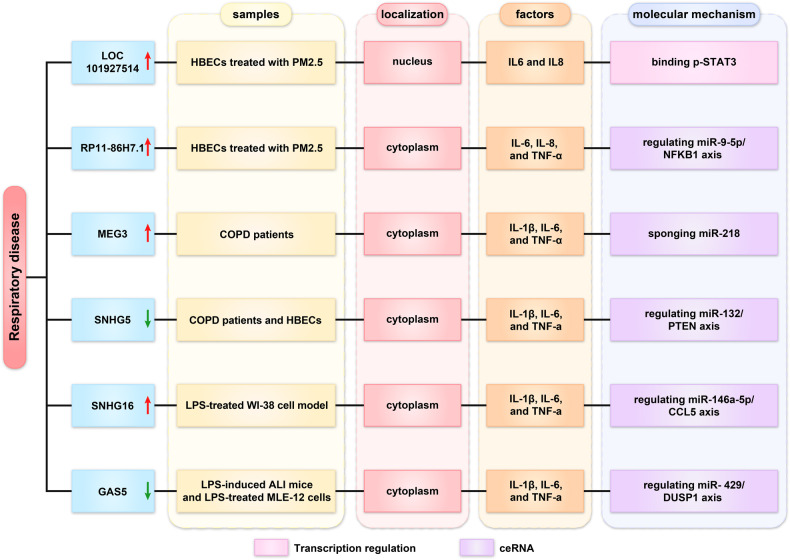
Roles of lncRNA in respiratory inflammatory disease.

Respiratory diseases are primarily caused by harmful gases and particles, such as particulate matter (PM2.5) and cigarette smoke extract (CSE). Chronic obstructive pulmonary disease (COPD), asthma, and pneumonia are common respiratory diseases partly caused by inflammatory responses [55]. Recent studies have shown that lncRNAs contribute to the development of respiratory diseases (Fig. 5).

### Regulation of transcription

Long-term exposures to PM2.5 can cause or aggravate respiratory tract inflammation. Tan et al. found that LOC101927514 was upregulated in human bronchial epithelial cells (HBECs) treated with PM2.5. The nucleus-localized LOC101927514 promoted the transcription of IL-6 and IL-8 by binding p-STAT3, thereby aggravating the inflammatory state of the cells [56].

### Regulation of miRNA sponge

LncRNA RP11-86H7.1 was also significantly upregulated in PM2.5-treated HBECs. RP11-86H7.1 could promote the inflammatory response by miR-9-5p/NFKB1 axis [57]. Additionally, lncRNA MEG3 was upregulated in COPD patients. MEG3 knockdown alleviated CSE-triggered apoptosis and inflammation (IL-1β, IL-6, and TNF-α) by targeting miR-218 [58]. While SNHG5 expression was low in COPD tissues. Overexpression of SNHG5 could weaken the effects of CSE on proliferation, apoptosis, and IL-1β, IL-6, and TNF-a levels in 16HBE cells via miR-132/PTEN axis [59]. Additionally, acute lung injury (ALI) is a life-threatening syndrome characterized by excessive inflammation and apoptosis of alveolar epithelial cells. SNHG16 was upregulated in the LPS-treated WI-38 cell model. SNHG16 could mediate the JNK and NF-κB pathways by the miR-146a-5p/CCL5 axis in acute pneumonia [60]. In contrast, lncGAS5 was downregulated in the lung tissues in LPS-induced acute lung injury (ALI) mice and LPS-treated MLE-12 cells. GAS5 suppresses inflammatory responses and apoptosis of alveolar epithelial cells by targeting miR-429/DUSP1 axis [61].

These data suggested that lncRNAs can regulate the expression of inflammatory factors and may represent a new therapeutic target for respiratory inflammatory diseases.

## Roles of lncRNAs in diabetic retinopathy

**Fig. 6:**
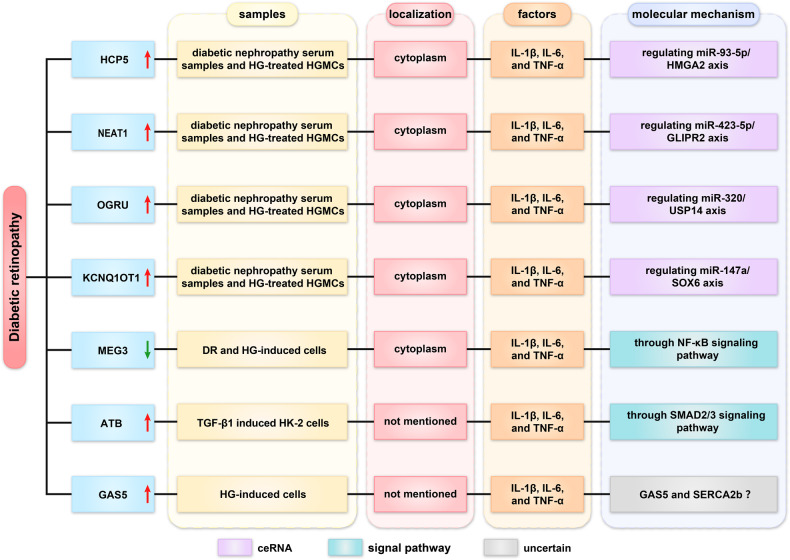
Roles of lncRNA in diabetic retinopathy disease.

Diabetic retinopathy (DR) is a serious complication of diabetes that can lead to blindness [62]. Inflammation and apoptosis are hallmarks of DR, but their regulatory mechanisms are poorly understood. Herein, we summarized the lncRNA regulatory mechanism in DR (Fig. 6).

### Regulation of miRNA sponge

High glucose (HG)-induced cells were frequently used to establish DN cell models. LncHCP5, lncNEAT1, lncOGRU, and lncKCNQ1OT1 were upregulated in serum samples of diabetic nephropathy and HG-treated HGMCs. HCP5 knockdown may weaken inflammation by modulating the miR-93-5p/HMGA2 [63]. NEAT1 knockdown may inhibit DN progression through the miR-423-5p/GLIPR2 axis [64]. Similarly, OGRU knockdown ameliorated DR progression via miR-320/USP14 [65]. KCNQ1OT1 knockdown suppressed proliferation, inflammation, and oxidative stress via the miR-147a/SOX6 axis [66].

### Regulation of signal pathway

HG may increase IL-1β, IL-6, and TNF-α levels in cells, whereas DR and high glucose (HG)-induced cells elicit a decrease in lncRNA MEG3. MEG3 overexpression can inhibit apoptosis and inflammatory response by inhibiting the NF-κB signaling pathway [67]. Moreover, transforming growth factor-β1 (TGF-β1) significantly contributes to renal fibrosis. TGF-β1 induced HK2 cells served as the cell model. LncATB was highly expressed in TGF-β1 induced HK2 cells. ATB knockdown may inhibit inflammation through the SMAD2/3 signaling pathway [68].

### Uncertain regulatory mechanisms

LncRNA GAS5 was highly expressed in HG‑treated cells. GAS5 may suppress apoptosis and inflammation by regulating SERCA2b. However, the specific mechanism of GAS5 requires further investigation [69]. These findings suggested that lncRNAs can regulate the expression of inflammatory factors and may represent a new therapeutic target for DR.

## Roles of lncRNAs in Parkinson’s disease

Parkinson’s disease (PD) is an age-related neurodegenerative disease [70]. The accumulated evidence confirms that lncRNA is involved in the progress of PD (Table 1).

**Table 1:** The mechanisms of lncRNA in Parkinson’s disease.

lncRNA	Expression level	Samples	Localization	Factors	Molecular mechanism	Reference
SNHG7	upregulation	serum of PD patients	cytoplasm	IL-1β, IL-6, and TNF-α	regulating miR-425-5p/TRAF5 axis	[71]
HOTAIR	upregulation	APP/PS1 mice	cytoplasm	IL-1β, IL-6, and TNF-α	sponging miR-130a-3p	[72]
MALAT1	upregulation	serum of PD patients	not mentioned	IL-1β, IL-6, and TNF-α	sponging miR155, miR124, or targeting NF-κB?	[73]
TUG1	upregulation	serum of PD patients	not mentioned	IL-1β, IL-6, and TNF-α	not mentioned	[76]

### Regulation of miRNA sponge

Serum SNHG7 levels were upregulated in PD patients. Downregulation of SNHG7 decreased IL-6, IL-1β, and TNF-α levels by regulating miR-425-5p/TRAF5/NF-KB signaling pathway [71]. Interestingly, physical activity contributed to the elevated expression of HOTAIR in APP/PS1 mice. HOTAIR inhibited the expression of IL-1β, IL-6, and TNF-α by targeting miR-130a-3p. This implies that moderate exercise can effectively reduce the symptoms of Alzheimer’s disease [72].

### Uncertain regulatory mechanisms

MALAT1 and TUG1 lncRNAs were upregulated in the serum of PD patients. MALAT1 could increase the secretion of IL-1β, IL-6, and TNF-α in LPS-treated PC12 cells and induce an inflammatory response [73]. The underlying mechanism may involve sponging miR155, miR124, or targeting NF-κB; however, more convincing evidence is required [74, 75]. Similarly, the downregulation of TUG1 significantly inhibited the expression of IL-6, IL-1β, and TNF-α and improved the motor coordination of PD mice, although the precise mechanism underlying TUG1 remains unknown [76]. The data suggested that lncRNAs are involved in the inflammatory response and may represent a potential therapeutic target. Additional potentially functional lncRNAs have yet to be identified in PD.

## Roles of lncRNAs in macrophage polarization

**Fig. 7:**
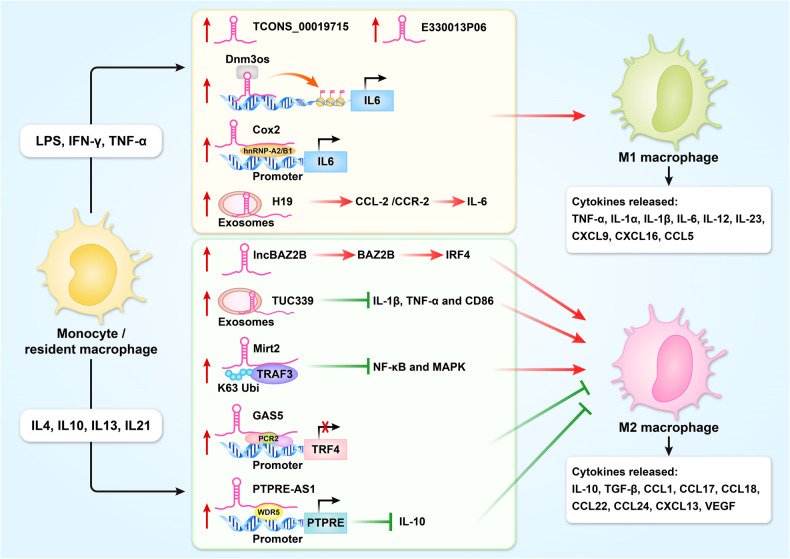
Roles of lncRNA in macrophage polarization.

In short, lncRNAs and inflammatory factors play important roles in the occurrence and development of human diseases, and the vast majority of inflammatory factors are secreted by macrophages. It is necessary to introduce how lncRNAs regulate the polarity of macrophages to secrete corresponding inflammatory factors. Macrophages are the fundamental inflammatory cells. In the early stages of tissue injury, macrophages initiate inflammation and manifest as an M1 type to remove exogenous threats. In the later stages of inflammation, M2 macrophages are polarized, recognize phosphatidylserine on apoptotic cells, eliminate apoptotic cells, and control inflammation [77–80]. Recent studies have found that lncRNAs are involved in the dynamic transformation of macrophages (Fig. 7).

### Regulation of transcription

LncRNA Dnm3os and Cox2 were upregulated in monocytes. By modulating histone H3K9-acetylation, overexpression of Dnm3os induces inflammation, M1 polarization, and immune-related gene expression [81]. Cox2 overexpression may enhance IL-6 level and several critical regulators of M1 polarization through interaction with hnRNP-A2/B1 [82]. However, GAS5 and PTPRE-AS1 were negatively associated with the polarization of M2 macrophages. GAS5 suppresses TRF4 transcription by recruiting the polycomb repressive complex 2 (PRC2), inhibiting M2 polarization in demyelinating diseases [83]. PTPRE-AS1 knockdown enhanced M2 macrophage activation by binding directly to WDR5 to modulate H3K4me3 of the PTPRE promoter [84].

### Regulation of mRNA stability

Li et al. found that lnc-BAZ2B was upregulated in monocytes and asthmatic children. Lnc-BAZ2B could promote the mRNA stability of BAZ2B and the transcription of IRF4, thereby promoting the activation of M2 macrophages in asthma [85].

### Regulation of signal pathway

Exosomes have emerged as important M1 polarization signaling mediators. Exosomal H19 significantly promotes the secretion of IL-6 through CCL-2/CCR-2 signaling pathways and enhances the activation of M1 polarization in Kupffer cells [86]. Du et al. found that Mirt2 was positively associated with M2 macrophage polarization. Mirt2 is associated with the ubiquitin-ligase TRAF6 and inhibits the activation of NF-κB and MAPK pathways, thus promoting M2 polarization [87].

### Uncertain regulatory mechanism

Huang et al. found that lncRNA TCONS_00019715 was upregulated in M1 macrophages and positively associated with the expression of M1 markers [88]. Additionally, lncRNA E330013P06 was upregulated in monocytes. E330013P06 overexpression may enhance inflammatory responses and induce M1 differentiation [89]. Moreover, exosomal lncRNA TUC339 was positively associated with M2 macrophage polarization. TUC339 knockdown leads to increased production of IL-1β, TNF-α, and CD86 and inhibits M2 polarization in THP-1 cells [90].

## Conclusions and perspectives

In recent years, lncRNAs have highlighted the significance of cellular functions such as stem cell maintenance, differentiation, apoptosis, cellular homeostasis, and the inflammatory process [91]. This paper summarized the expression level and key roles of lncRNAs in inflammatory diseases. Firstly, lncRNA expression levels are also closely related to inflammatory diseases. For example, atherosclerosis was positively associated with the high expression of lncRNA-CCL2, lncANRIL, and lncRNA ENST00000416361. High expression levels of lncDLEU1, LOXL1-AS1, and LINC00265 were positively associated with osteoarthritis. High expression levels of lncRNAs UCA1 and CRNDE were positively associated with sepsis. Liu et al. also found that lncRNA H19, LINC00895, lnc-SRGAP2C-16, lnc-HLA-C-2, lnc-APOC1-1, and lnc-B3GALT2-1 were associated with the progression of chronic non-atrophic gastritis [92]. Ma et al. revealed that MIAT promoted allergic inflammation in mice with allergic rhinitis [93]. Liu et al. also found that NEAT1 knockdown may attenuate LPS-induced inflammation and apoptosis in HMEECs [94]. He et al. observed that H19 could promote keratinocyte proliferation and inflammation in psoriasis [95]. Tian et al. also identified that lncRNA CDKN2B-AS1 regulated inflammation of ulcerative colitis [96]. These results suggested that lncRNAs have a promising future as novel biomarkers for inflammatory diseases. LncRNAs involved in vaginitis, cervicitis, shoulder periarthritis, etc., have been rarely reported. However, these inflammatory diseases also harm human health, necessitating urgent research on lncRNA. Moreover, lncRNAs are also involved in various cancer types. For example, PCA3 and PCGEM1 are highly specific to prostate cancer [97, 98]. HOTAIR, ANRIL, MALAT1, and LNP1 were positively associated with breast cancer [99]. HNF1A-AS1, ANRIL, and H19 were positively associated with lung cancer [100, 101]. These findings implied that lncRNAs might serve as a new marker for cancer diagnosis. An increasing amount of experimental data confirms that lncRNAs are associated with cancer, and applications are on the horizon.

LncRNAs may regulate the release of inflammatory cytokines, the activation of the cell signaling pathways, and the activation of immune cells in inflammatory disease. The exact mechanism of lncRNAs primarily involves transcription and post-transcriptional regulation, including chromatin modification, mRNA degradation, and miRNA sponging. The most prevalent method is miRNA sponging. For example, MALAT1/miR-590, NEAT1/miR-342-3p, DLEU1/miR-671-5p, LOXL1-AS1/miR-423-5p, and LINC00265/miR-101-3p axes may regulate inflammatory gene expression and subsequently participate in the development of related diseases. Ma et al. found that lncRNA-associated ceRNA networks could facilitate the diagnosis and treatment of Alzheimer’s disease [102]. Additionally, Zheng et al. found that lncRNA GAS5-mediated ceRNA regulatory pathways may represent a novel insight and a potential research direction for heart failure [103]. These findings support the need for future research to find new lncRNA mechanisms.

Inflammatory diseases pose a grave threat to human health and life, and their incidence is declining [104]. It is necessary to develop lncRNA-based treatments for inflammatory diseases. There are some promising applications of lncRNAs in the prognosis and treatment of inflammatory diseases. Firstly, lncRNAs can be used as early diagnostic indicators or treatment response markers [105]. HOTAIR, GAS5, and HIX003209 have been identified as promising novel biomarkers for RA [106]. Secondly, lncRNAs could be used as a therapeutic strategy in inflammation-related diseases by artificially manipulating the disease-related lncRNA level. Such as the efficient delivery of microparticles coated with si-Neat1, resulting in a significantly improved osteolysis effect [107]. Moreover, kaempferol is a flavonoid compound with diverse biological activities, such as antioxidant, anticancer, and anti-inflammatory properties. The ability of kaempferol to weaken XIST expression and then inhibit inflammation and extracellular matrix degradation in chondrocytes implies that siRNA may replace conventional drugs in clinical settings [108]. However, unlike protein-coding genes, lncRNAs are poorly conserved across different species. The clinical significance of these lncRNAs has not been completely established. Most of these measurements were conducted between humans and animals and have not been used in clinical research. The clinical application of lncRNAs requires further development.
